# Human pan-cancer analysis of the predictive biomarker for the CDKN3

**DOI:** 10.1186/s40001-024-01869-6

**Published:** 2024-05-08

**Authors:** Yingjun Chen, Dai Li, Kaihui Sha, Xuezhong Zhang, Tonggang Liu

**Affiliations:** 1 Department of Infectious Diseases, Binzhou Medical University Hospital, Binzhou, 256600 Shandong China; 2 Department of General Surgery, The Fourth Affiliated Hospital of China Medical University, Shenyang, 110000 Liaoning China; 3 Binzhou Medical University School of Nursing, Binzhou, 256600 Shandong China; 4 Department of Laboratory Medicine, Zibo Central Hospital, Zibo, 255000 Shandong China

## Abstract

**Background:**

Cell cycle protein-dependent kinase inhibitor protein 3 (CDKN3), as a member of the protein kinase family, has been demonstrated to exhibit oncogenic properties in several tumors. However, there are no pan-carcinogenic analyses for CDKN3.

**Methods:**

Using bioinformatics tools such as The Cancer Genome Atlas (TCGA) and the UCSC Xena database, a comprehensive pan-cancer analysis of CDKN3 was conducted. The inverstigation encompassed the examination of CDKN3 function actoss 33 different kinds of tumors, as well as the exploration of gene expressions, survival prognosis status, clinical significance, DNA methylation, immune infiltration, and associated signal pathways.

**Results:**

CDKN3 was significantly upregulated in most of tumors and correlated with overall survival (OS) of patients. Methylation levels of CDKN3 differed significantly between tumors and normal tissues. In addition, infiltration of CD4 + T cells, cancer-associated fibroblasts, macrophages, and endothelial cells were associated with CDKN3 expression in various tumors. Mechanistically, CDKN3 was associated with P53, PI3K-AKT, cell cycle checkpoints, mitotic spindle checkpoint, and chromosome maintenance.

**Conclusion:**

Our pan-cancer analysis conducted in the study provides a comprehensive understanding of the involvement of CDKN3 gene in tumorigenesis. The findings suggest that targeting CDKN3 may potentially lead to novel therapeutic strategies for the treatment of tumors.

**Supplementary Information:**

The online version contains supplementary material available at 10.1186/s40001-024-01869-6.

## Introduction

Cancer stands as one of the most formidable challenges to global human health [1]. Despite considerable researches efforts, the intricacies of tumor evolution remain elusive [2]. Conducting comprehensive pan-cancer analyses, rather than focusing solely on individual tumors, holds promise in elucidating tumor pathogenesis. This approach not only facilitates advancements in treatment modalities but also mitigates the risk of drug resistance development [3].

CDKN3, a member of the protein kinase family, is pivotal in cell cycle regulation, primarily through its influence on ubiquitination-mediated protein degradation [4, 5]. Additionally, it interacts with phosphatase KAP, exerting regulatory control over cell cycle progression [6–9]. As a crucial factor in cellular regulation, CDKN3 has been demonstrated to promote matrix degradation and inflammatory response in coronary artery endothelial cells [10–12]. Recently several studies reported that CDKN3 played a important role in the inflammatory response observed in severe cases of COVID-19 [13–15]. Furthermore, CDKN3 has been implicated in tumor progression. Studies have established a notable upregulation of CDKN3 in lung cancer [16], gastric cancer [17] and breast cancer [18] correlating significantly with poor patient prognosis. Subsequent investigations have validated that CDKN3 exerts influence on the drug resistance of bladder cancer cells by disrupting glycolysis, thereby impacting metabolic reprogramming [19]. Similar effects on drug resistance have also been substantiated in colorectal cancer and liver cancer [20–23]. However, researches on CDKN3 has been limited to a few cancer types, leaving its role in other cancers ambiguous.

In our study, we examined the expression of CDKN3 across various types of cancer using a pan-cancer analysis encompassing multiple databases. We analyzed several factors, including gene expressions, survival prognosis, genetic alterations, DNA methylation, immune infiltration levels and gene enrichment analysis. This comprehensive approach aimed to elucidate the potential molecular mechanisms underlying the involvement of CDKN3 in cancer pathogenesis or clinical prognosis.

## Materials and methods

### Gene expression analysis

The clinical data of this study is from the TCGA (http://portal.gdc.cancer.gov/) and the UCSC Xena database (https://xenabrowser.net/datapages/). We obtained relevant RNA seq data through the UCSC XENA database. We use log2 conversion to analyze RNA seq data in TPM format. And use R software to analyze the data. We also use the R software package "ggplot2" for visualization. We also used UALCAN software to detect the differences in gene expression levels of CDKN3 at different stages in tumor and normal tissues. The P-value threshold is 0.05.

### Survival prognosis analysis

We obtained clinical information on CDKN3 patients through the TCGA database. We conducted relevant prognostic analysis based on indicators such as OS, disease-specific survival (DSS), and progression-free interval (PFI). We also evaluated the survival probability of different tumor patients through univariate Cox regression analysis and Kaplan Meier survival analysis. The relevant data is analyzed using R packets. We also used timeROC to assess the predictive ability of CDKN3 as a clinical indicator.

### Relationship between CDKN3 expression and clinical features

Exploring the association between the expression of CDKN3 and relevant clinical indicators (gender, pathological stage, and TNM staging) using R packets. The relevant data was analyzed using the ggplot2 software package.

### Establishment and evaluation of the nomogram models

Analyze which tumors may have an impact on the prognosis of CDKN3. The univariate Cox regression analysis was performed on the relevant tumors. To create a column chart model, select tumors with statistical significance and a sampling size above 500. And use calibration curves to determine the accuracy of the 1-, 3-, and 5-year column charts.

### Immune infiltration analysis

The relationship between CDKN3 expression and immune infiltration was analyzed through TIMER2 online. And immune cells such as T cells, macrophages, and fibroblasts were selected as reference objects. Evaluate the degree of immune infiltration using quantitative methods such as TIDE, XCELL, and EPIC. Use the purity-adjusted Spearman test to calculate P-values and correlation (cor) results. The data obtained above is presented through a heat map.

### Methylation analysis

Use the UALCAN website to detect methylation differences between tumors and normal tissues. And generate relevant data using the TCGA dataset.

### Gene enrichment analysis and protein–protein interaction (PPI) network analysis

We used the GEPIA2 database to obtain the 100 genes most closely associated with CDKN3 (Additional file 1: Table S1). Analyze the function of CDKN3 through GO analysis and KEGG pathway analysis. In addition, we generated a PPI network using 100 CDKN3-related genes on the STRING website (Additional file 1).

### Gene set enrichment analysis

We conducted GSEA analysis using differential expression of CDKN3. And attempt to elucidate the biological function of CDKN3 in tumor progression.

## Results

### CDKN3 expression in pan-cancer

**Fig. 1:**
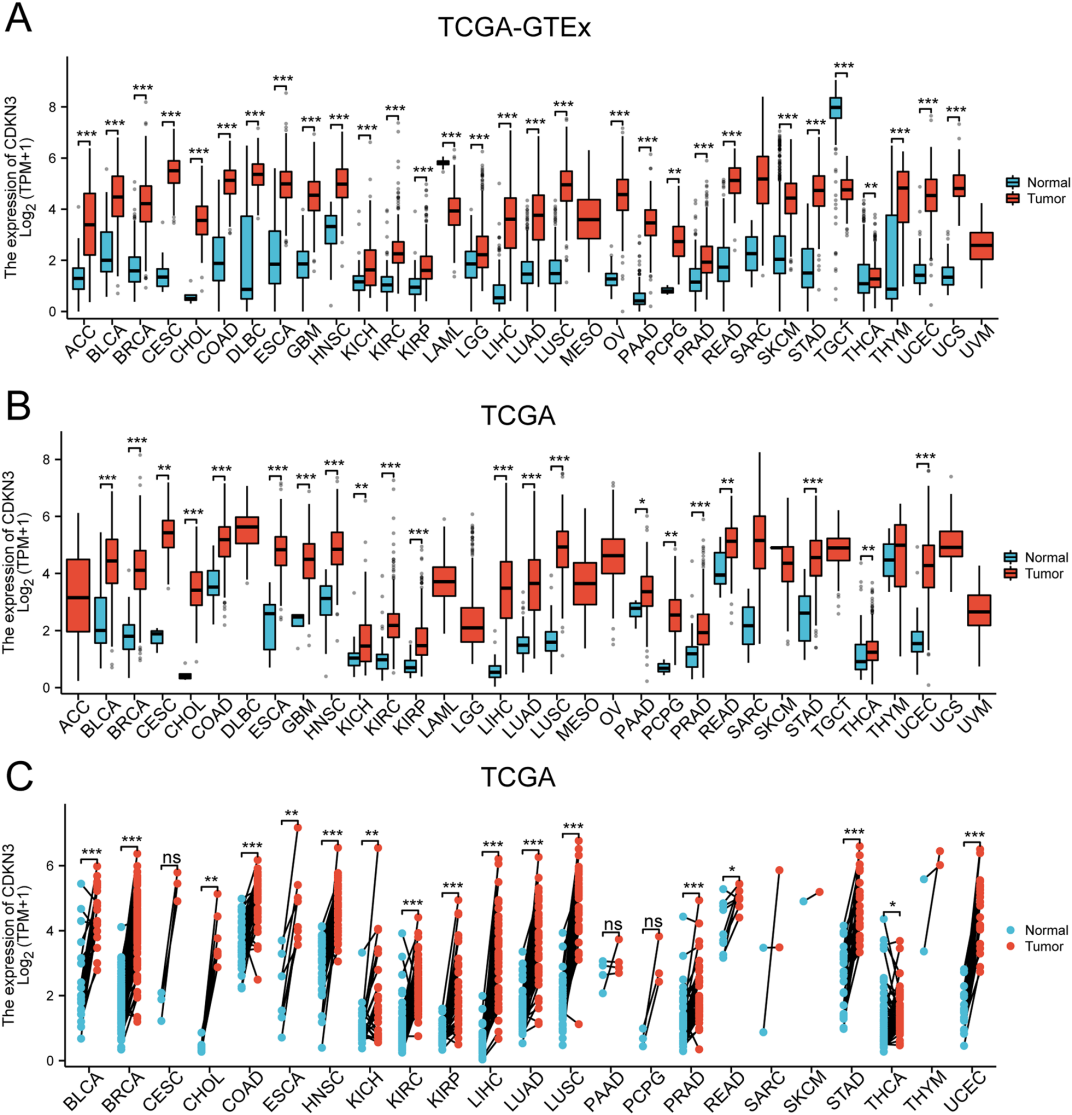
CDKN3 mRNA expression in pan-cancers. **A** CDKN3 mRNA expression in 33 tumors from TCGA-GTEx samples. **B** CDKN3 mRNA expression in 33 tumors from the TCGA database. **C** CDKN3 expression was observed in 23 paired tumor specimens from the TCGA database. (ns, p ≥ 0.05; *p < 0.05; **p < 0.01;***p < 0.001)

In this study, we conducted an analysis of TCGA_GTEx data obtained from UCSC to explore the expression of CDKN3 in pan-cancer. Our investigation unveiled diverse expression patterns of the CDKN3 gene within distinct tumor cells. tumor cells. The expression of CDKN3 was significantly up-regulated in majority of tumors. However, CDKN3 expression was significantly down-regulated in LAML and TGCT (Fig. 1A). We also found that the expression of CDKN3 was significantly overexpressed in most of tumors. This result harmoniously resonated with the observations gleaned from the TCGA dataset (Fig. 1B). We also scrutinized CDKN3 expression in both tumors and corresponding normal tissues. Intriguingly, barring THCA, a consistent trend emerged wherein the majority of tumor tissues demonstrated heightened CDKN3 expression relative to their corresponding normal tissue counterparts. However, there was no conspicuous change in CDKN3 expression was observed between the normal tissues and the tumor tissues of CESC, PAAD, and PCPG (Fig. 1C).

Furthermore, we also obtained alterations in CDKN3 expression levels at distinct tumor stages by the utilization of the UALCAN online tool. In the advanced stages of 16 diverse cancer types, including BLCA, BRCA, CESC, COAD, ESCA, CHOL, KICH, KIRC, KIRP, HNSC, LIHC, LUAD, LUSC, READ, STAD, and UCEC, we observed a significant augmentation in CDKN3 expression (Additional file 1: Fig. S1).

### The association between CDKN3 expression and prognosis in pan-cancer

**Fig. 2:**
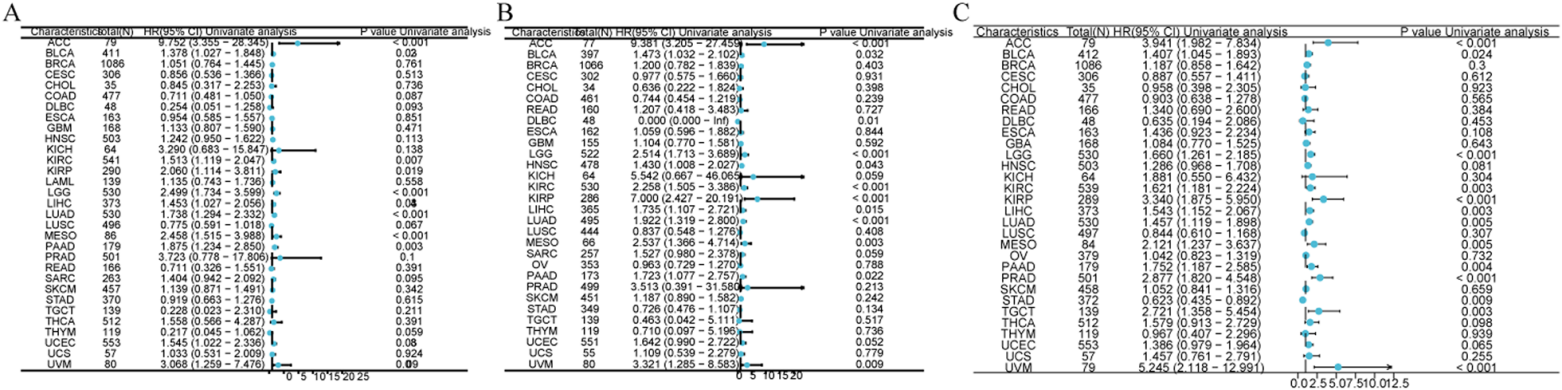
CDKN3 expression correlates with OS, DSS, and PFIin pan-cancer survival analysis. **A** Forest plots of the OS survival results. **B** Forest plots of the DSS survival results. **C** Forest plots of the PFI survival results

Kaplan–Meier survival analysis was used to investigate the correlation between CDKN3 expression and clinical outcomes. As shown in Fig. 2A, we delved further into the association between CDKN3 expression and overall survival (OS) in 33 distinct cancers. The results demonstrated a compelling correlation between abnormal CDKN3 expression and OS in a subset of cancers, including ACC, BLCA, KIRC, KIRP, LGG, LIHC, LUSC, MESO, PAAD, UCEC, and UVM (Additional file 1: Fig. S2A–K). Importantly, it was discerned that high level of CDKN3 was associated with shorter OS in these particular cancer types.

Subsequently, we explored the association between CDKN3 expression and disease specific survival (DSS) (Fig. 2B). Our exploration yielded compelling insights, revealing distinct associations between CDKN3 expression and DSS in a range of cancer types. Notably, the findings showed significant associations between CDKN3 expression and DSS in ACC, BLCA, DLBC, LGG, HNSC, KIRC, KIRP, LIHC, LUAD, MESO, PAAD, and UVM (Additional file 1: Fig. S3A–L). In these specific cancers, elevated CDKN3 expression was conspicuously correlated with poorer DSS.

Finally, an in-depth exploration into the relationship between CDKN3 expression and Progression-Free Interval (PFI) was undertaken (Fig. 2C). This endeavor yielded noteworthy findings, enabling us to discern a clear pattern where elevated CDKN3 expression aligns with adverse PFI outcomes across several tissue types. Specifically, our analysis revealed that high CDKN3 expression is indicative of poorer PFI in the following tissues: ACC, BLCA, LGG, KIRC, KIRP, LIHC, LUAD, MESO, PAAD, PRAD, STAD, TGCT, and UVM (Additional file 1: Fig. S4A–M). Additional file 1: Fig. S5A–E showcases Receiver Operating Characteristic (ROC) curves for five tumors where the prognosis is notably linked to CDKN3 expression, effectively illustrating the diagnostic potential of CDKN3 in these cases.

### The relationships between CDKN3 expression and clinical parameters

The expression of CDKN3 was related to the prognosis of 17 different types of tumors, including ACC, BLCA, DLBC, HNSC, LGG, KIRC, KIRP, LIHC, LUAD, LUSC, MESO, PAAD, UCEC, PRAD, STAD, TGCT, and UVM. Here, we investigated the relationships between CDKN3 expression and the clinicopathological characteristics of these 17 tumors. These findings revealed that in the cases of HNSC, KIRP, LUAD, and LUSC, CDKN3 expression was associated with gender (Additional file 1: Fig. 3A–D). In the meantime, tumor size exhibited a connection with CDKN3 expression in ACC, KIRC, KIRP, and LIHC (Additional file 1: Fig. 3E–H). Additionally, CDKN3 expression was associated with lymph node metastases in HNSC, KIRC, KIRP, LUAD, LUSC, and PRAD (Additional file 1: Fig. 3I–N). There was also a correlation between CDKN3 expression and the pathological stage in ACC, KIRC, KIRP, LIHC, LUAD, and LUSC (Additional file 1: Fig. 3O–T).

**Fig. 3:**
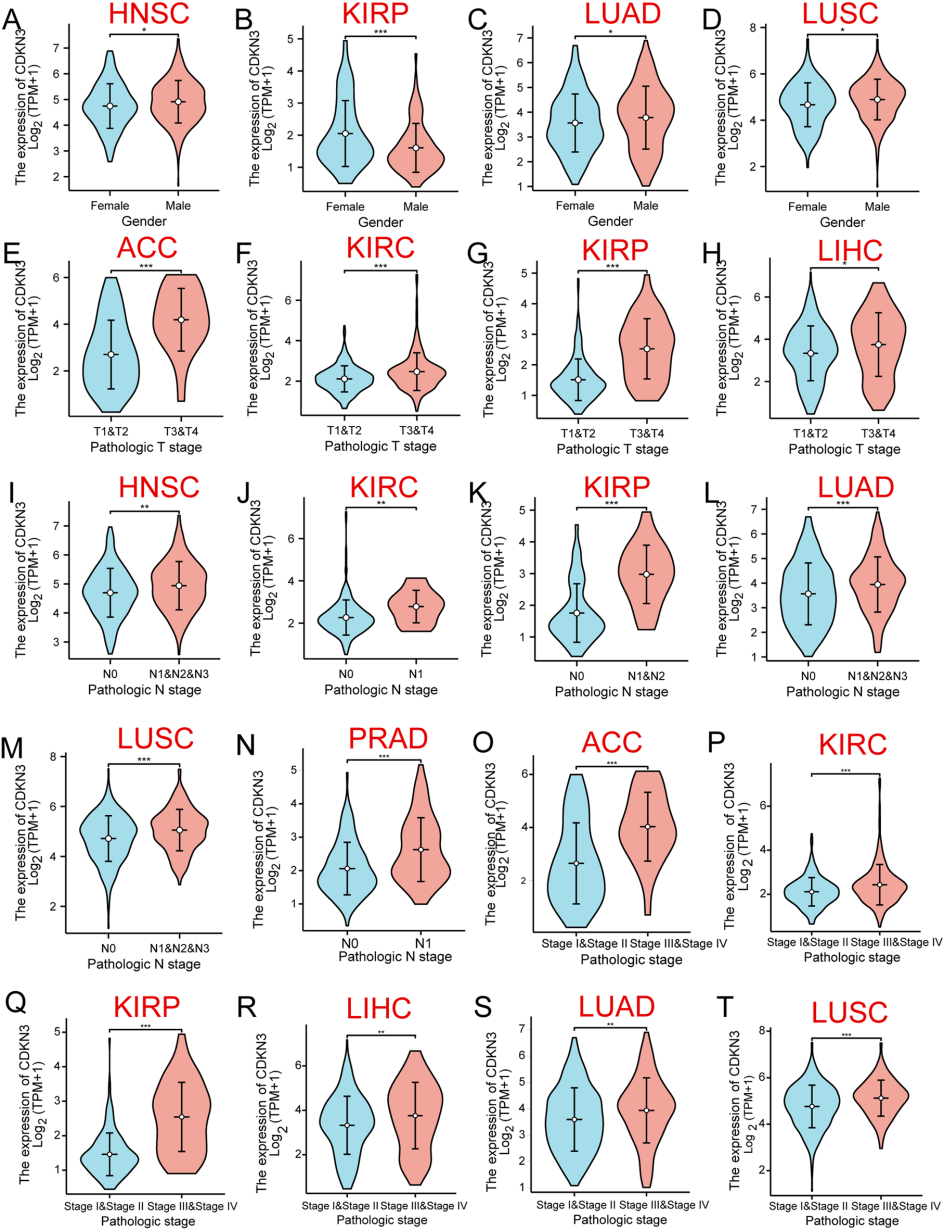
Clinical metrics and CDKN3 expression's relationship **A**–**D** CDKN3 expression was related to gender. **E**–**H** Expression of CDKN3 was related to the T stage. **I**–**N** CDKN3 expression was related to the N stage. **O**–**T** CDKN3 expression was related to pathologic stage. (*p < 0.05; **p < 0.01; ***p < 0.001)

### Building and assessing nomogram models for kidney renal clear cell carcinoma and lung squamous cell carcinoma

In order to investigate the effect of CDKN3 expression on the prognosis of certain tumors, we performed univariate Cox regression analysis for OS in nine tumors (Additional file 1: Tables S2–S10). To evaluate the prognostic value, we employed calibration curves to assess the prediction accuracy of nomogram model across 1, 3 and 5-year periods. KIRC and LIHC with sample sizes more than 400 were selected. These models were constructed using the findings of a single-variate Cox regression. Results indicated that CDKN3 had a significant capacity to predict OS for KIRC and LIHC (Additional file 1: Fig. 4A,C), and calibrated survival prediction curves at 1, 3 and 5-year demonstrated that the nomogram model had a high level of precision and accuracy (Additional file 1: Fig. 4B, D).

**Fig. 4:**
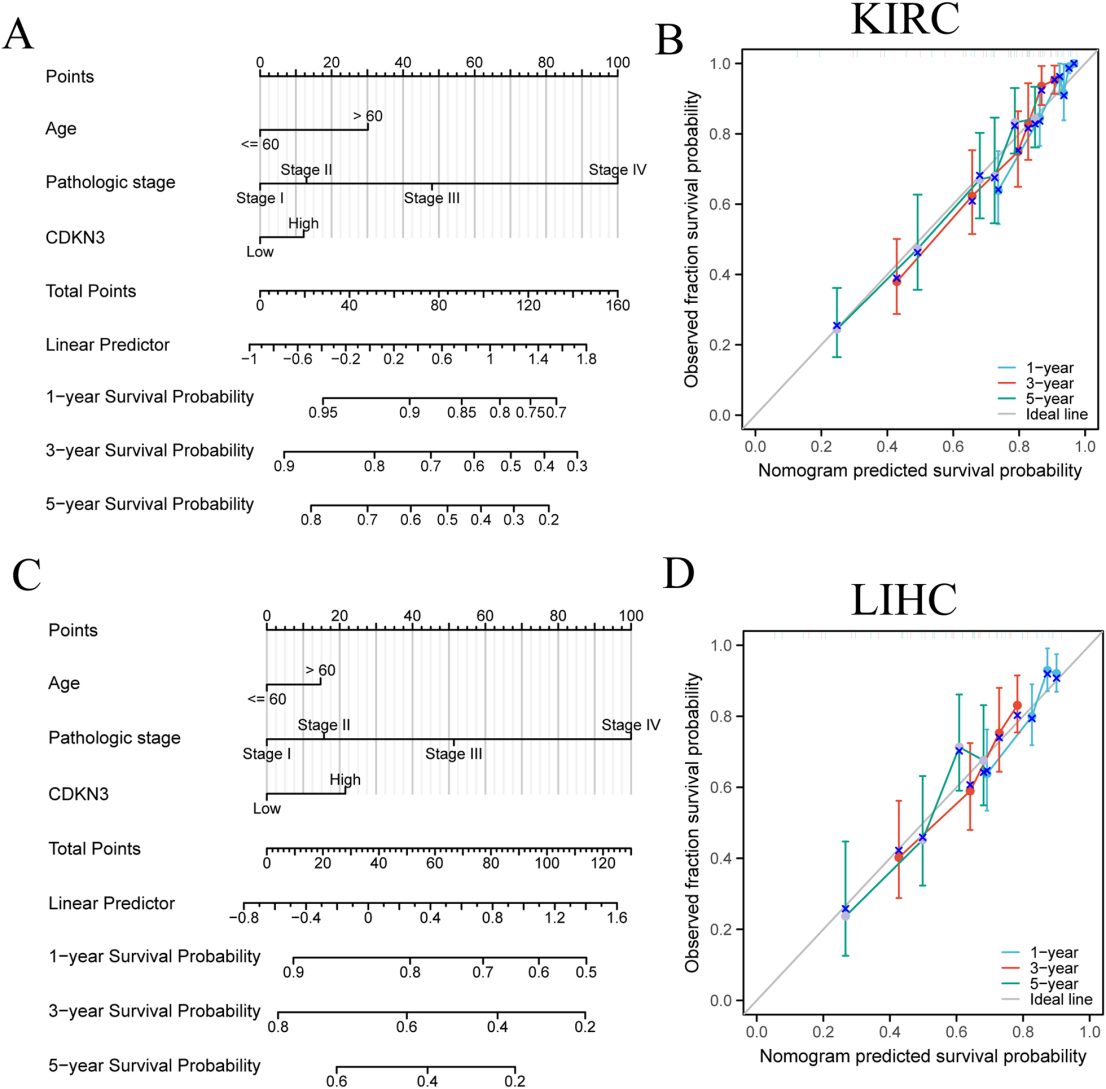
The CDKN3 prognostic signature was combined with independent TCGA components to create our hybrid nomogram. **A** Creation of a nomogram model that takes CDKN3 expression in KIRC into account. **B** Using calibration curves for 1, 3, and 5 years, we assessed the KIRC nomogram model's prediction accuracy. C The expression of CDKN3 in LIHC is modelled using a nomogram. **D** Using calibration curves for 1, 3, and 5 years, we assessed the LIHC nomogram model's prediction accuracy

### The correlation of CDKN3 expression and tumor immune microenvironment

**Fig. 5:**
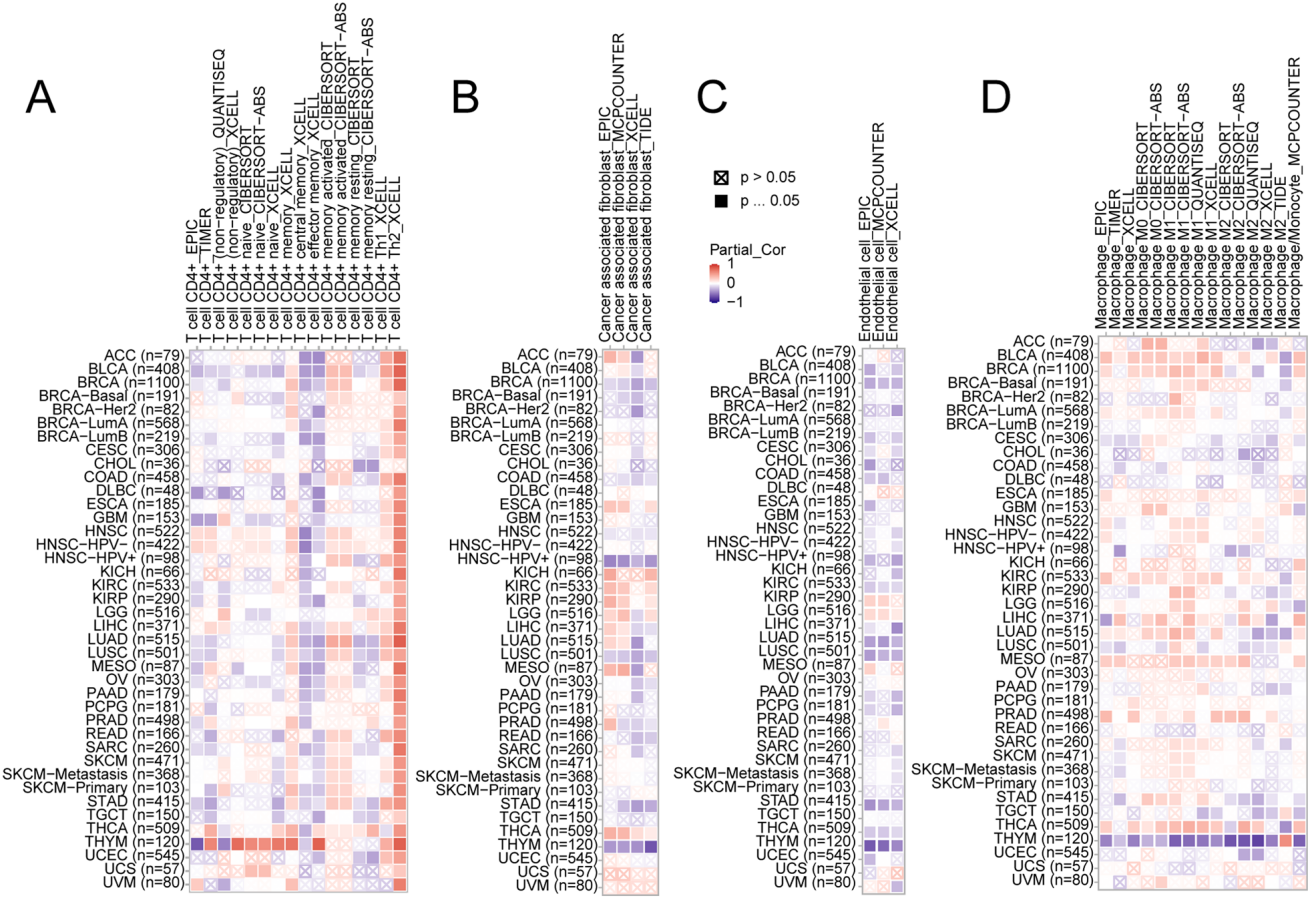
Analysis of the relationship between CDKN3 expression and immune infiltration of CD4 + T cells (**A**), Cancer associated fibroblasts (**B**), endothelial cells (**C**), and Macrophages (**D**)

The progression of tumors are significantly influenced by the immune microenvironment. To investigate the relationship between CDKN3 and the immune microenvironment in pan-cancer, we conducted an analysis using the GEPIA2 database to assess the correlation between CDKN3 expression and immune cells. Heatmaps were used to illustrate the associations between CDKN3 expression and CD4 + T cells, cancer-associated fibroblasts, macrophages, and endothelial cells (Fig. 5A–D). Over-expression of CDKN3 was significantly associated with Th2 (Fig. 5A). In the TCGA tumors of BRCA, HNSC-HPV + , LUSC, and THYM tumors, we found a statistically significant negative connection between CDKN3 expression and infiltrating cancer-associated fibroblasts. However, CDKN3 expression in THCA was positively connected with fibroblast infiltration related to malignancy (Fig. 5B). Furthermore, we found a statistically significant inverse relationship between CDKN3 expression and endothelial cells in the BRCA, KIRC, LUAD, LUSC, STAD, and THYM tumors. The expression of CDKN3 was positively linked with endothelial cells in LGG (Fig. 5C). Figure 5D demonstrated significant correlations between macrophages and CDKN3 expression in BLCA, KIRC,HNSC-HPV-, MESO, PRAD, and THCA.

### DNA methylation analysis

**Fig. 6:**
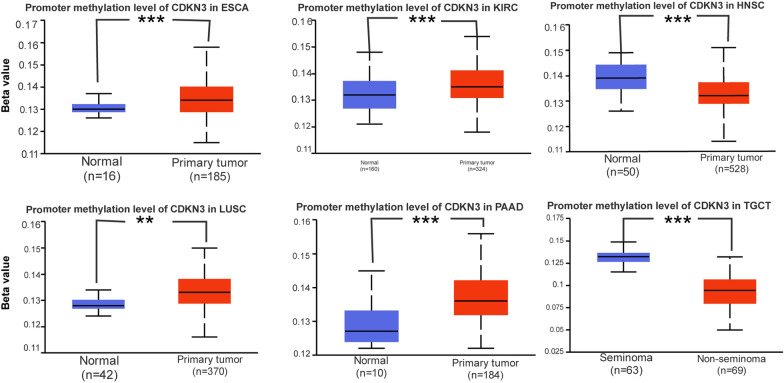
The methylation level of CDKN3 in ESCA, KIRC, HNSC, LUSC, TGCT, and PAAD

Tumor development, growth, and cellular carcinogenesis are all tightly connected with abnormal DNA methylation. The degree of DNA methylation of certain genes as well as variations in DNA methylation levels may also be used to detect tumors [24]. Using the UALCAN and TCGA databases, the DNA methylation levels of CDKN3 between normal and primary tumor tissues were explored. The CDKN3 methylation expression levels in HNSC and TGCT tumor tissues were significantly down-regulated (Fig. 6). Additionally, ESCA, KIRC, LUSC, and PAAD tumor tissues had considerably higher levels of CDKN3 methylation expression (Fig. 6).

### Functional enrichment and protein–protein interactions of CDKN3-related genes

**Fig. 7:**
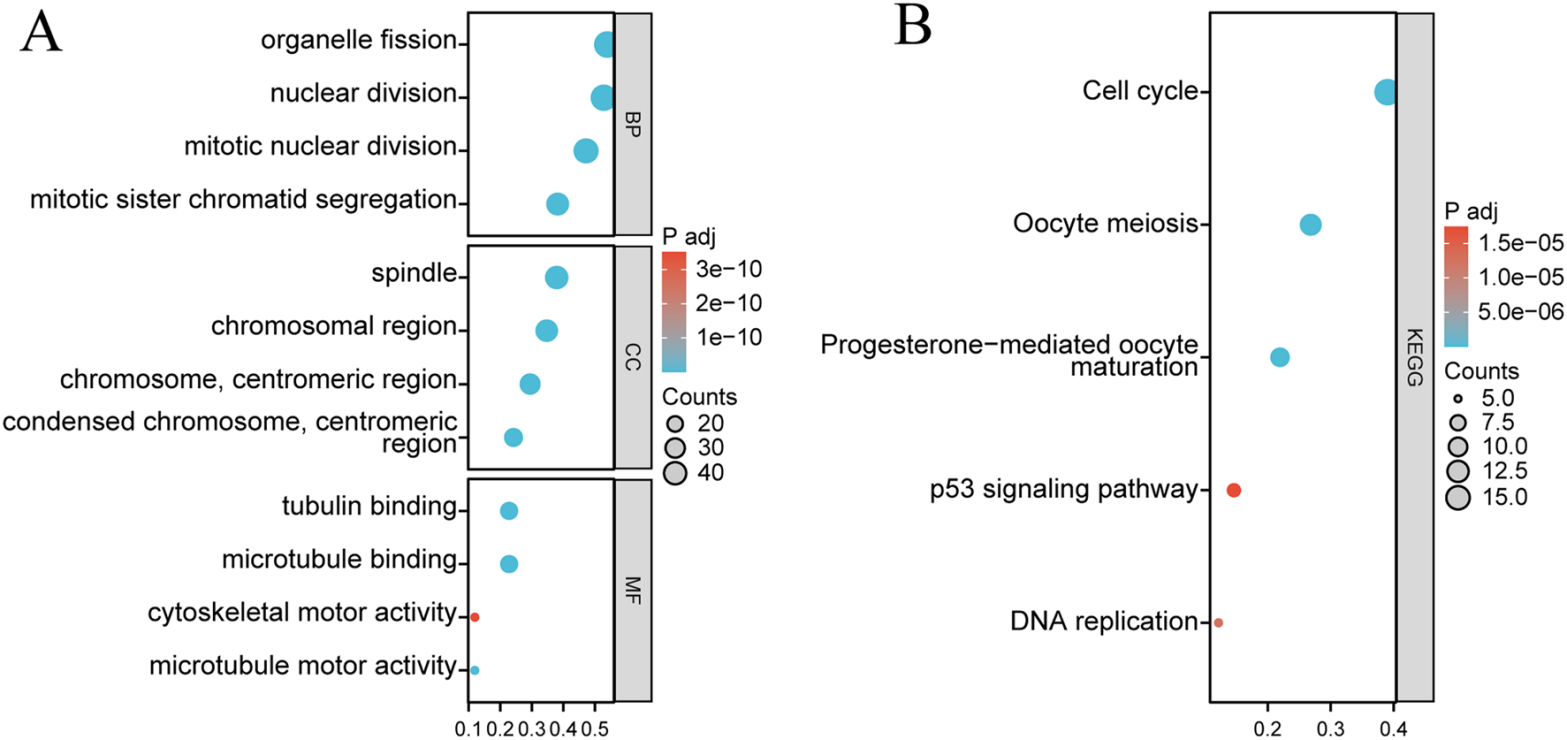
Analysis of CDKN3-related genes' functional enrichment. **A** Analyses of GO functional enrichment (BP, CC, and MF). **B** Analysis of KEGG pathways for 100 CDKN3-related genes

From the GEPIA2 database, 100 genes with the closest relationships to CDKN3 were analyzed to better understand the biological role of CDKN3 in tumors (Additional file 1: Table S1). According to GO analysis (Fig. 7A), CDKN3-related genes may be involved in a variety of biological processes, including "mitotic sister chromatid segregation," "organelle fission," "nuclear division," and "mitotic nuclear division." Involved in “spindle”,“chromosomal region”, “chromosome, centromeric region”and other cell components. Along with other molecular activities, it takes part in "microtubule binding," "tubulin binding," and "microtubule motor activity." CDKN3-related genes may be related to "Cell cycle," "Oocyte meiosis," "Progesterone-mediated oocyte maturation," "DNA replication," and "p53 signalling pathway," according to KEGG pathway analysis (Fig. 7B). The PPI network on the STRING website was constructed using 100 CDKN3-related genes (Additional file 1: Fig. S6). Collectively, these analyses provided a comprehensive framework for understanding the biological significance of CDKN3 in the context of tumors, unraveling its involvement in vital cellular processes, molecular interactions, and pathways that influence tumor development and progression.

### Gene set enrichment analysis

**Fig. 8:**
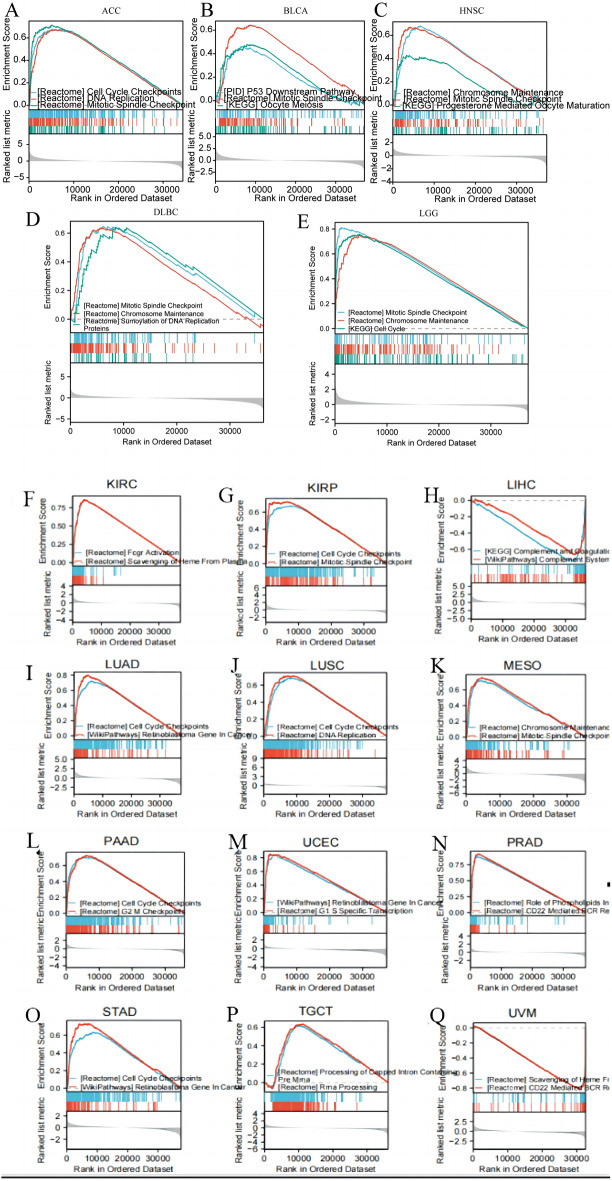
CDKN3-related genes' GSEA analysis. **A**–**Q** GSEA based on differential expression analyses for ACC, BLCA, HNSC, DLBC, LGG, KIRC, KIRP, LIHC, LUAD, LUSC, MESO, PAAD, UCEC, PRAD, STAD, TGCT and UVM

The GSEA analysis was used to clarify the biological function of CDKN3 in the 17 tumors with CDKN3 related to prognosis. These 17 tumors included ACC, BLCA, HNSC, DLBC, LGG, KIRC, KIRP, LIHC, LUAD, LUSC, MESO, PAAD, UCEC, PRAD, STAD, TGCT, and UVM (Fig. 8A–Q). The findings imply that CDKN3 was primarily involved in mitotic spindle checkpoints, cell cycle checkpoints, and chromosome maintenance.

## Discussion

The presence of tumor heterogeneity diminishes treatment efficacy and contributes to poor prognosis. Despite advancements in understanding tumor cell subpopulations facilitated by emerging technologies like single-cell sequencing, progress in clinical oncology remains sluggish. Hence, it is crucial to expedite clinical translation by identifying novel tumor markers.

Currently, significant progress has been made in CDKN3 research, highlighting its pivotal role in regulating cell cycle progression. Further investigations have revealed its involvement in severe COVID-19, female reproductive toxicity, mitotic control, and adipocyte proliferation. Moreover, the role of CDKN3 in the tumor microenvironment has begun to emerge. Extensive studies on its regulation of individual tumor progression indirectly suggest its potential as a tumor marker target. However, there remains a dearth of research analyzing the suitability of CDKN3 as a tumor marker from a macro perspective.

In this study, we conducted pan-cancer analysis using bioinformatics data. We observed significant variations in CDKN3 expression between tumor and normal tissues across TCGA GTEx samples, TCGA samples, and TCGA paired samples. With the exception of THCA, we noted higher CDKN3 expression in most tumor tissues compared to paired normal tissues. However, we encountered inconsistencies in certain aspects. For instance, the conclusions derived from TCGA_GTEx and THCA data were diametrically opposite to those from TCGA data. We attribute these disparities to differences in the sample size of the control group. Therefore, to enhance result accuracy, we recommend augmenting the sample size of the control group to mitigate the likelihood of such discrepancies.

There is currently no comprehensive study evaluating the prognostic value of CDKN3 in various cancers. In our study, we elucidated the multifaceted prognostic implications of CDKN3 overexpression on tumor overall survival (OS) based on analyses of TCGA and GEO databases. Our findings suggest that elevated CDKN3 expression correlates with poorer OS, disease-specific survival (DSS), disease-free interval (DFI), and progression-free interval (PFI), particularly in cancers such as ACC, BLCA, KIRC, KIRP, LGG, LIHC, MESO, PAAD, and UVM. Moreover, additional investigations have validated that increased CDKN3 expression promotes proliferation and metastasis of renal cell carcinoma [25]. Upon reviewing relevant databases, we noted a dearth of research exploring the prognostic implications of CDKN3 in bladder tumors, multiple myeloma, neuroendocrine tumors, and melanoma [26]. Consequently, we expanded our study to include these tumor types. Our results underscore a significant association between CDKN3 expression and prognosis in these unexplored malignancies. Thus, future research efforts can focus on unraveling the underlying mechanisms driving this correlation to deepen our understanding of tumor evolution.

Interestingly, we found some connections between CDKN3 and immune cells. In the tumor microenvironment, immune cells play an extremely important role as soil for tumors. Our analysis indicates a certain correlation between CDKN3 and CD4 + T cells, fibroblasts, macrophages, and endothelial cells. The levels of CDKN3 and macrophage infiltration vary among different types of cancer. Therefore, we grouped them based on the level of CDKN3 expression. Through grouping, we attempted to explore the interaction between CDKN3, immune cells, and tumor prognosis. Based on these findings, we found a strong correlation between CDKN3 and the degree of immune cell infiltration. Conversely, high levels of immune cell infiltration indicate poor tumor prognosis. Therefore, we speculate that high levels of CDKN3 may interfere with the prognosis of tumor patients by affecting immune cells. This is consistent with many previous research findings [27, 28].

The relationship between CDKN3 and clinical prognosis is not limited to immune cells in the tumor microenvironment. In addition to the prognosis and CDKN3 expression of 17 types of tumors analyzed by TCGA, we also found a correlation between CDKN3 expression and tumor size in ACC, KIRC, KIRP, and LIHC. It is worth considering whether CDKN3 is related to certain aspects of tumor cell renewal and proliferation? Research has shown that miR-127-3p promotes proliferation and metastasis of renal cell carcinoma through CDKN25. ZNF677 also inhibits the progression of renal cell carcinoma through the transcription of N6 methyladenosine and CDKN [29]. In addition, CDKN3 has been shown to be an independent prognostic factor that contributes to the progression of nasopharyngeal carcinoma to advanced stage [30]. This is consistent with our conclusion. In addition to tumor progression, we also found that CDKN3 is associated with lymph node metastasis in six types of tumors. This result has also been similarly confirmed in oral cancer [31]. In summary, we can confirm that the presence of CDKN3 predicts poor tumor prognosis. Therefore, using CDKN3 as a tumor treatment target will be very important. There have been studies using transcriptomics to determine that CDKN3 is a core gene for the prognosis of colorectal cancer [32]. We believe that CDKN3 will have broader research prospects in the future.

In current research on tumors, in addition to the immune microenvironment and various common cell death modes (such as apoptosis, autophagy, etc.) mentioned above, epigenetic modification is one of the current research hotspots. Methylation modification has always been a hot research topic for many scholars. Our study also found that the methylation expression level of CDKN3 in HNSC and TGCT tumor tissues is significantly lower than that in normal tissues. In addition, the methylation expression level of CDKN3 was significantly increased in ESCA, KIRC, LUSC, and PAAD tumor tissues. This proves that CDKN3 may be positively correlated with methylation. So, is there a correlation between CDKN3 and methylation? It has been proven that ZNF677 inhibits the progression of renal cell carcinoma through the transcription of N6 methyladenosine and CDKN3 [29]. It also indicates that the regulation of neuroblastoma cell proliferation can alter the methylation of the CDKN3 gene promoter region [33]. Interestingly, CDKN3, as an RNA methylation related isomer of pancreatic cancer, has been proved to be involved in immune infiltration. This can enable us to determine whether CDKN3 is associated with methylation, which may be related to the correlation we discussed in the previous paragraph. This further reflects the fact that in the tumor microenvironment, information exchange between cells manifests as a network structure, in which CDKN3 plays a crucial role.

Through the analysis of this article, we found a strong correlation between CDKN3 and the P53 and PI3K-AKT pathways, and related studies have also confirmed this point [34, 35]. However, through our research, we found that the NOTCH signaling pathway is also associated with CDKN3, but this has not been confirmed in relevant studies. It is worth pondering why there is currently no research focusing on this signaling pathway? After all, the NOTCH signaling pathway has been proven to be a key hub for maintaining tumor cell stemness and causing DNA mutations. Is it because the relationship between NOTCH and CDKN3 is not as close as we analyzed, or is it because of sample size, tumor type, or statistical errors that have led to bottlenecks in related research? This is the focus we can explore in the future.

In addition to the clarifications mentioned above, we recognize the importance of leveraging current advancements and technological changes in the era of big data for clinical research. As artificial intelligence technology continues to evolve, our capacity to analyze large-scale datasets will substantially enhance. Moreover, integrating advanced imaging technology with increasingly sophisticated genomics will furnish more objective insights for future research endeavors. Therefore, it's imperative for us to direct our attention towards advancements at the forefront of technology [36].

The research emphasized the role of CDKN3 in pan cancer analysis. Through different analyses, we provide examples to demonstrate the feasibility of CDKN3 as a tumor marker. However, there were several limitations. This study did not validate the significance of CDKN3 through relevant experimental methods. Additionally, we aspire to incorporate information from single-cell sequencing libraries in future research endeavors to achieve a more detailed classification of tumor cells. We firmly believe that conducting a more comprehensive analysis of the relationship between CDKN3 and the tumor microenvironment holds immense significance for the development of targeted therapies for tumors.

## Conclusions

In summary, we conducted a comprehensive pan cancer study on CDKN3. We observed the significant relationship between CDKN3 expression and clinical prognosis, gene mutation, DNA methylation, immune cell infiltration and tumor mutation in a variety of human malignant tumors, trying to help understand the function of CDKN3 in tumors from multiple perspectives.

### Supplementary Information

**Additional file 1.** Supplementaryfigures and tables.

## Data Availability

The datasets used in this investigation are available through public repositories.

## Abbreviations

CDKN3: Cell cycle protein-dependent kinase inhibitor protein 3
TCGA: The Cancer Genome Atlas
OS: Overall survival
CPTAC: Clinical Proteomics tumor Analysis Consortium
DSS: Disease-specific survival
PFI: Progression-free interval
cor: Correlation
ACC: Adrenocortical carcinoma
BLCA: Bladder urothelial carcinoma
LGG: Lower grade glioma
HNSC: Head and neck squamous cell carcinoma
KIRC: Kidney clear cell carcinoma
KIRP: Kidney papillary cell carcinoma
LIHC: Liver cancer
LUSC: Lung squamous cell carcinoma
MESO: Mesothelioma
PAAD: Pancreatic cancer
UVM: Uveal melanoma
ROC: Receiver operating characteristic
